# Accumulation of DNA Damage-Induced Chromatin Alterations in Tissue-Specific Stem Cells: The Driving Force of Aging?

**DOI:** 10.1371/journal.pone.0063932

**Published:** 2013-05-17

**Authors:** Nadine Schuler, Claudia E. Rübe

**Affiliations:** Department of Radiation Oncology, Saarland University, Homburg/Saar, Germany; Università di Milano, Italy

## Abstract

Accumulation of DNA damage leading to stem cell exhaustion has been proposed to be a principal mechanism of aging. Using 53BP1-foci as a marker for DNA double-strand breaks (DSBs), hair follicle stem cells (HFSCs) in mouse epidermis were analyzed for age-related DNA damage response (DDR). We observed increasing amounts of 53BP1-foci during the natural aging process independent of telomere shortening and after protracted low-dose radiation, suggesting substantial accumulation of DSBs in HFSCs. Electron microscopy combined with immunogold-labeling showed multiple small 53BP1 clusters diffusely distributed throughout the highly compacted heterochromatin of aged HFSCs, but single large 53BP1 clusters in irradiated HFSCs. These remaining 53BP1 clusters did not colocalize with core components of non-homologous end-joining, but with heterochromatic histone modifications. Based on these results we hypothesize that these lesions were not persistently unrepaired DSBs, but may reflect chromatin rearrangements caused by the repair or misrepair of DSBs. Flow cytometry showed increased activation of repair proteins and damage-induced chromatin modifications, triggering apoptosis and cellular senescence in irradiated, but not in aged HFSCs. These results suggest that accumulation of DNA damage-induced chromatin alterations, whose structural dimensions reflect the complexity of the initial genotoxic insult, may lead to different DDR events, ultimately determining the biological outcome of HFSCs. Collectively, our findings support the hypothesis that aging might be largely the remit of structural changes to chromatin potentially leading to epigenetically induced transcriptional deregulation.

## Introduction

The maintenance and propagation of accurate genetic information is critical in ensuring proper cellular functions and in preventing cancer formation or aging [1]. In highly regenerative organs, such as skin epidermis, homeostasis is maintained by tissue-specific stem cells that reside in protective microenvironments where they generate functional differentiated cells that replenish lost or damaged cells throughout the lifetime of the organism [2]. Stem cells are at high risk of accumulating deleterious DNA lesions because they are so long-lived. Such damage may limit the survival or functionality of the stem cell population and may even initiate or promote carcinogenesis. Furthermore, accumulation of short or damaged telomeres elicit a DNA damage response (DDR), are considered as a main source of aging-associated DNA damage and may cause the exhaustion of the proliferative potential of stem and progenitor cells [3], [4].

DNA lesions arise from both endogenous chemical reactions, such as reactive oxygen species (ROS) generated by cellular metabolism, and exogenous insults coming from the surrounding environment, such as ionizing radiation. Double-strand breaks (DSBs) are the most dangerous lesions because misrepaired or unrepaired DSBs can lead to genomic instability and cell death. Cells respond to DSBs by activating DDR mechanisms that recognize DNA damage, repair the lesions, and, if the damage is too extensive, stimulate cells to undergo either programmed cell death (apoptosis) or irreversible cell cycle arrest (senescence). Non-homologous end-joining (NHEJ) is the main DSB repair pathway in G0/G1 cells and upon formation of a DSB, each broken terminus binds a Ku70/Ku80 heterodimer that then associate to form a bridging complex that recruits additional NHEJ factors, including DNA-PKcs and DNA ligase IV/XRCC4. After the final ligation reaction, the Ku molecules are released from the rejoined DNA [5], [6].

The organization of DNA into chromatin has a major influence on the cellular response to DNA damage [7], [8]. In eukaryotic cells, DNA is tightly associated with histones and non-histone proteins to form the higher order chromatin structure. DNA methylation and histone modifications, including lysine methylation and acetylation, regulate the chromatin accessibility and distinct transcriptional functions. Epigenetic hallmarks of silenced or heterochromatic DNA include histone 3 lysine 9 and lysine 27 tri-methylation (H3K9me3; H3K27me3), and histone 4 lysine 20 di−/tri-methylation (H4K20me2/3). Open or active euchromatic DNA is associated with acetylated histones such as acetylation of histone 3 lysine 9 (H3K9ac) or histone 4 lysine 16 (H4K16ac), as well as methylation of histone 3 at lysine 4 (H3K4me) [9]. The ability of repair factors to detect DNA lesions and be retained efficiently at breaks is determined by histone modifications around the DSBs and involves chromatin-remodeling events that facilitate repair by promoting chromatin accessibility. 53BP1 is rapidly recruited to regions of chromatin next to the DNA break and forms nuclear foci. The visualization of 53BP1-foci by immunofluorescence microscopy allows to quantify DSBs and elucidate DNA damage signaling and repair pathways, even in complex normal tissues [10], [11], [12], [13]. Although 53BP1 is not a core NHEJ component, it has been shown to function in several NHEJ-dependent rejoining events such as radiation-induced DSB repair and telomere fusions at deprotected telomeres [14].

We have established a transmission electron microscopy (TEM) approach to detect gold-labeled repair components in different chromatin environments. The ultra-high resolution of TEM offers the intriguing possibility of detecting core components of the DNA repair machinery at the single-molecule level and visualizing their molecular interactions with specific histone modifications. We showed that damage-response proteins such as γH2AX, MDC1, and 53BP1 that can be found in foci after radiation exposure assemble exclusively at heterochromatin-associated DSBs. By labeling phosphorylated Ku70 (pKu70), which binds directly to broken DNA ends in preparation for rejoining, this TEM approach can monitor formation and repair of DSBs in euchromatic and heterochromatic regions [15], [16].

Oxidative stress is considered to be the major contributor to the aging process, and damage caused by ROS predominantly leads to single-strand break formation. However, DSBs can also arise when ROS-induced lesions are encountered by the replication or transcription machinery, or when they arise in close proximity to each other. Oxidative DNA damage continuously occurs throughout the lifespan of the organism and appears to contribute to the accumulation of DSBs in heterochromatic DNA regions [17], [18]. Accumulation of DNA damage foci has been described in various tissues of aged animals and even in hematopoietic stem and progenitor cells during human aging [19], [20], [21].

Limited information is available about how tissue-specific stem cells activate DDR pathways in response to genotoxic insults within their physiological microenvironment. Here, using mouse epidermis of aging animals as an in-vivo model, we analyzed the molecular events of the DDR in epidermal stem cells in the bulge region located in the upper part of the outer root sheath of the hair follicle. These hair follicle stem cells (HFSCs), which can easily be identified in hairy mouse skin by the stem cell marker CD34, represent the most potent reserve population, capable of regenerating all skin cell types [22]. They are pluripotent and have considerable proliferative potential, but spend most of their time in a quiescent G1/G0 state, dividing only 2–5 times in one hair cycle [2].

Quantifying individual foci as markers for DNA lesions by immunofluorescence microscopy, we analyzed the age-related DSB repair capacity of HFSCs within their physiological environment. We monitored DNA damage accumulation during the natural aging process and in the course of repeated exposure to low doses of ionizing radiation. Combining immunofluorescence with fluorescence in-situ hybridization (FISH), we studied the role of telomere shortening in relation to age-associated DNA damage accumulation. Analyzing HFSCs at the ultrastructural level by TEM, we characterized the persistent DNA lesions induced by intrinsic DNA damage during chronological aging or extrinsic DNA damage triggered by protracted low-dose radiation. Using flow-assisted cytometry, we studied the molecular events of the DDR induced by these genotoxic insults and correlated these functional changes to the biological outcome of HFSCs. The aim of this study was to better define the fundamental molecular mechanisms that control the aging process.

## Materials and Methods

### Animals

C57BL/6 and SCID mice were purchased from Charles River Laboratories (Sulzfeld, Germany). The mice were housed four to six per cage in pathogen-free rooms (temperature 22±2°C, humidity 55±10%, 12 h light-dark cycle) to minimize the risk for infections and supplied with standard laboratory diet and water ad libitum. Serological assessment was conducted at least quarterly to test for infection, and all tests were negative.

### Radiation Schedule and Tissue Sampling

C57BL/6 and SCID mice of different ages (0.5 to 24 months) received whole-body radiation at a linear accelerator (Artiste™, Siemens). The mice were placed in an 18 cm diameter plexiglass cylinder covered by 1.5 cm thick plastic material to improve photon dose homogeneity. The radiation characteristics were as follows: size of the radiation field, 30 cm×30 cm; collimator angle 0°; gantry angle 0°; source surface distance, 208 cm; beam energy, 6 MV photons; dose-rate, 2 Gy/min. Computed-tomography-based 3-dimensional dose calculations were made with the Pinnacle™ planning system (Philips Radiation Oncology Systems, Fitchburg, WI). For evaluating the repair of DSBs after single-dose radiation, three C57BL/6 mice per dose were analyzed 0.5 h, 5 h, 24 h, and 48 h after exposure to 2 Gy. For evaluating the effects of daily low-dose radiation with 10 mGy, mice were irradiated once daily from Monday to Friday with 24 h between exposures. After single-dose exposure as well as 2, 4, 6, or 8 weeks of radiation, animals were anesthetized and the dorsal skin of the back was removed 24 h and 72 h after the last exposure, fixed, and processed for further analysis. The experimental protocol was approved by the Medical Sciences Animal Care and Use Committee of the University of Saarland.

### Immunofluorescence Analysis

Formalin-fixed tissues were embedded in paraffin and sectioned at a thickness of 4 µm. After dewaxing in xylene and rehydration in decreasing concentrations of alcohol, sections were boiled in citrate buffer and incubated with Roti™-Immunoblock (Carl Roth, Karlsruhe, Germany). Sections were incubated with primary antibodies (anti-53BP1 and anti-γH2AX, Bethyl Laboratories, Montgomery, USA; anti-CD34, BD Biosciences, Heidelberg, Germany; anti-active Caspase-3, R&D Systems, Minneapolis, USA), followed by AlexaFluor-488 or AlexaFluor-568 secondary antibodies (Invitrogen, Karlsruhe, Germany). Finally, sections were mounted in VECTAshield™ with 4′,6-diamidino-2-phenylindole (DAPI; Vector Laboratories, Burlingame, USA). For quantitative analysis, 53BP1-foci were counted visually under a Nikon E600 epifluorescent microscope (Nikon, Düsseldorf, Germany). Counting of 53BP1-foci was performed until at least 40 foci and at least 40 cells were registered for each skin sample. Accordingly, in aged or low-dose irradiated animals with very low foci levels per cell we analyzed 200–400 cells in skin tissue of each mouse, and averaged the results obtained from three different mice for each data point. For quantitative analysis of apoptosis, 300 CD34^+^ HFSCs and 500 epidermal cells were screened for co-localization with Caspase-3 in the skin of three different young (2-month-old), aged (24-month-old) and low-dose irradiated mice (40× 10 mGy; 72 h).

### Simultaneous Immunofluorescence/FISH Analysis

After dewaxing and antigen retrieval, tissue sections were washed briefly in 96% ethanol. Sections were coated with PNA probe (800 ng/ml, Panagene Inc., Daejeon, Korea) diluted in hybridization buffer, denatured for 5 min at 80°C, and incubated for 2 h at 37°C. After hybridization, sections were washed and incubated with blocking buffer for 30 min, and the immunofluorescence staining for 53BP1 was performed as described above. To determine the percentage of colocalization, the number of 53BP1-foci colocalizing with fluorescent telomeric spots were counted in HFSCs and epidermal cells of 3- and 24-month-old mice. For every data point, at least 50 foci were analyzed for colocalization in tissue sections from three different mice.

### TEM Analysis

Tissue samples were cut into 2 mm^3^ cubes and fixed overnight with paraformaldehyde and glutaraldehyde. Fixed samples were dehydrated in increasing concentrations of alcohol and infiltrated with LR Gold resin™ (EMS, Hatfield, USA). Samples were then embedded in fresh resin with benzyl (EMS) and polymerized with ultraviolet light. Ultrathin sections were cut on an Ultracut UCT Leica™ with diamond knives (Diatome; Biel, Switzerland), picked up with pioloform-coated nickel grids, and processed for immuno-labeling. To block unspecific staining, sections were floated on glycine followed by blocking solution (EMS). Sections were then incubated with the primary antibody (anti-53BP1, anti-γH2AX (pSer139), Bethyl Laboratories; anti-pKu70 (pSer6), anti-pDNA-PKcs, Novus Biologicals, Littleton, USA; anti-H3K9me3, Abcam Inc., Cambridge, USA; anti-CD34, BD Biosciences). After rinsing, secondary antibody conjugated with 6-nm or 10-nm gold-particles (EMS) was applied to the grids. Sections were rinsed and post-fixed with glutaraldehyde. All sections were stained with uranylacetate and examined using a Tecnai Biotwin™ transmission electron microscope (FEI Company, Eindhoven, The Netherlands).

### Histochemical Detection of Senescence

After pre-fixation, skin samples were incubated in X-Gal staining solution (pH6), followed by end-fixation overnight [23]. Formalin-fixed and SA-β-gal-stained tissues were embedded in paraffin and sectioned at a thickness of 7 µm. After dewaxing in xylene and rehydration in decreasing concentrations of alcohol, antigen retrieval was performed in citrate buffer, and sections were incubated with anti-CD34 (BD Biosciences) or anti-p16^ink4a^ antibody (Cell Applications Inc., San Diego, USA) followed by a biotin-labeled antibody (Dako, Glostrup, Denmark). Staining was completed by incubation with 3,3′-diaminobenzidine and substrate chromogen. Finally, sections were counterstained with haematoxylin and mounted with Aqueous Mounting Medium (Dako). For quantitative analysis of senescence, 300 CD34^+^ HFSCs and 500 epidermal cells were screened for co-localization with SA-ß-gal in the skin of three different young (2-month-old), aged (24-month-old) and low-dose irradiated mice (40× 10 mGy; 72 h).

### FACS Analysis

Single-cell suspensions were harvested from the skin of three mice for every data point as previously described [24]. Cells were washed in 1% PBS-FBS and incubated with anti-CD34 antibody (BD Bioscience) in 1% PBS-FBS for 30 min. After washing, cells were incubated with anti-rat-APC secondary antibody (Molecular Probes, Life Technologies GmbH, Darmstadt, Germany) for 30 min and fixed in 2% formaldehyde for 15 min. Cells were permeabilized with 0.1% TritonX-100 (Sigma-Aldrich, St. Louis, USA) followed by 0.5% TritonX-100 diluted in PBS-FBS and incubated with anti-53BP1, anti-p53BP1, anti-γH2AX (Bethyl Laboratories), anti-pKu70, anti-H4K16ac, anti-H3K9ac, anti-H3K9me3 (Abcam Inc.), anti-pDNA-PKcs (Novus Biologicals), anti-Caspase-3 (R&D Systems), or anti-p16^ink4a^ (Cell Applications) antibody diluted in 0.1% TritonX-100. Cells were washed and incubated with the secondary IgGF(ab)_2_-APC-Cy7 antibody (Santa Cruz Biotechnology, Santa Cruz, USA). Following nuclear staining, the second stem cell marker anti-alpha 6-Integrin tagged with FITC (Abcam Inc.) was incubated for 30 min. Finally, cells were washed and diluted in 1% PBS-FBS for flow cytometry analysis. Each experiment was performed with unstained mock controls and with single staining of cell surface markers (CD34, alpha 6-Integrin). For quantitative analysis, around 30000 cells were gated by FSC and SSC and divided into CD34^+^ and CD34^–^ cells by CD34-APC and alpha 6-Integrin-FITC dot blot. These two populations were analyzed for their nuclear staining intensity of APC-Cy7. The maximal pixel and integrated fluorescence intensities were measured and recorded for each cell. Comparisons between the different study groups were made by the median of the measured APC-Cy7 fluorescence intensities.

### Statistical Analysis

To evaluate the potential differences between the different data groups, a one side Mann-Whitney-Test was performed using the statistical software OriginPro Software (version 8.5, OriginLab Corporation, Northampton, USA). The criterion for statistical significance was p≤ 0.05.

## Results

### 53BP1-foci as Markers for DSBs in HFSCs

**Figure 1:**
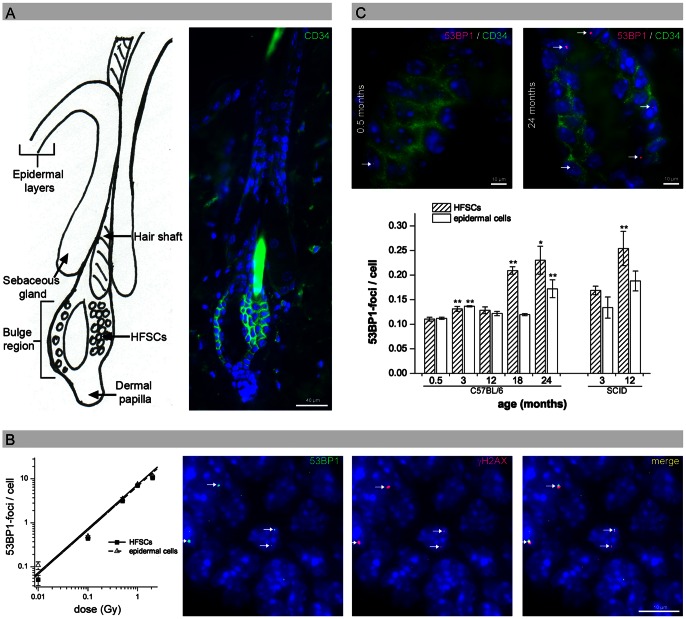
Age-related DNA damage accumulation in HFSCs. (A) IFM micrograph of CD34^+^ HFSCs (green) in the bulge region, in comparison to the schematic representation of the hair follicle. (B) Quantification of 53BP1-foci in HFSCs and epidermal cells analyzed 0.5 h after irradiation with different doses. IFM micrograph of radiation-induced 53BP1-foci in HFSCs, consistently colocalizing with γH2AX (0.5 h after 100 mGy). (C) Quantification of HFSCs and epidermal cells with spontaneous 53BP1-foci in repair-proficient C57BL/6 mice aged 0.5, 3, 12, 18, 24 months compared to repair-deficient SCID mice aged 3 and 12 months. Data are presented as means from three different experiments ±SE. * significant difference to 0.5-month-old mice; ** significant difference to the younger age-group. (Note: The hair shaft itself and hair-shaft cells display autofluorescence due to the keratin and melanin.).

Slow-cycling HFSCs can be identified within their local tissue microenvironment using specific cell surface markers such as CD34 (Fig. 1A). To establish reliable measurements of DNA damage in HFSCs, we performed CD34/53BP1 double-staining in mouse skin exposed to different doses of ionizing radiation and quantified the 53BP1-foci per cell in CD34^+^ HFSCs and epidermal cells (with a lifespan of 10–14 days) [25] (Fig.1B). Figure 1B shows that induction of 53BP1-foci in both HFSCs and epidermal cells depends on the radiation dose, with a linear correlation even after very low doses (Fig. 1B, left panel). These radiation-induced 53BP1-foci consistently colocalize with other markers for DSBs such as γH2AX, suggesting that the foci can be used to monitor unrepaired DSBs in HFSCs and epidermal cells in mouse epidermis (Fig. 1B, right panel).

### Age-related DNA Damage Accumulation in HFSCs

To quantify DNA damage accumulation during the physiological aging process, we measured the level of spontaneous 53BP1-foci in HFSCs and epidermal cells in repair-proficient C57BL/6 mice aged 0.5, 3, 12, 18, or 24 months (Fig. 1C, upper panel; Fig. S1). In 0.5-months-old mice, we observed 0.11 53BP1-foci per HFSC and 0.11 per epidermal cell. In 3- and 12-month-old mice we observed a slightly higher amount of 53BP1-foci, but between 3 and 12 months we observed no clear increase suggesting that DNA damage does not accumulate considerably during this time period. The skin of 18- and 24-month-old animals shows large numbers of foci, particularly in HFSCs (0.21±0.008 foci per cell at 18 months and 0.23±0.028 foci per cell at 24 months). Repair-deficient 12-month-old severe combined immunodeficiency (SCID) mice show even higher DNA damage levels, with 0.25±0.035 foci per HFSC (Fig. 1C, lower panel). These results demonstrate that 53BP1-foci accumulate in long-living HFSCs during aging, likely representing DSBs arising from endogenously induced ROS damage.

### Colocalization of 53BP1-foci with Telomeric DNA

**Figure 2:**
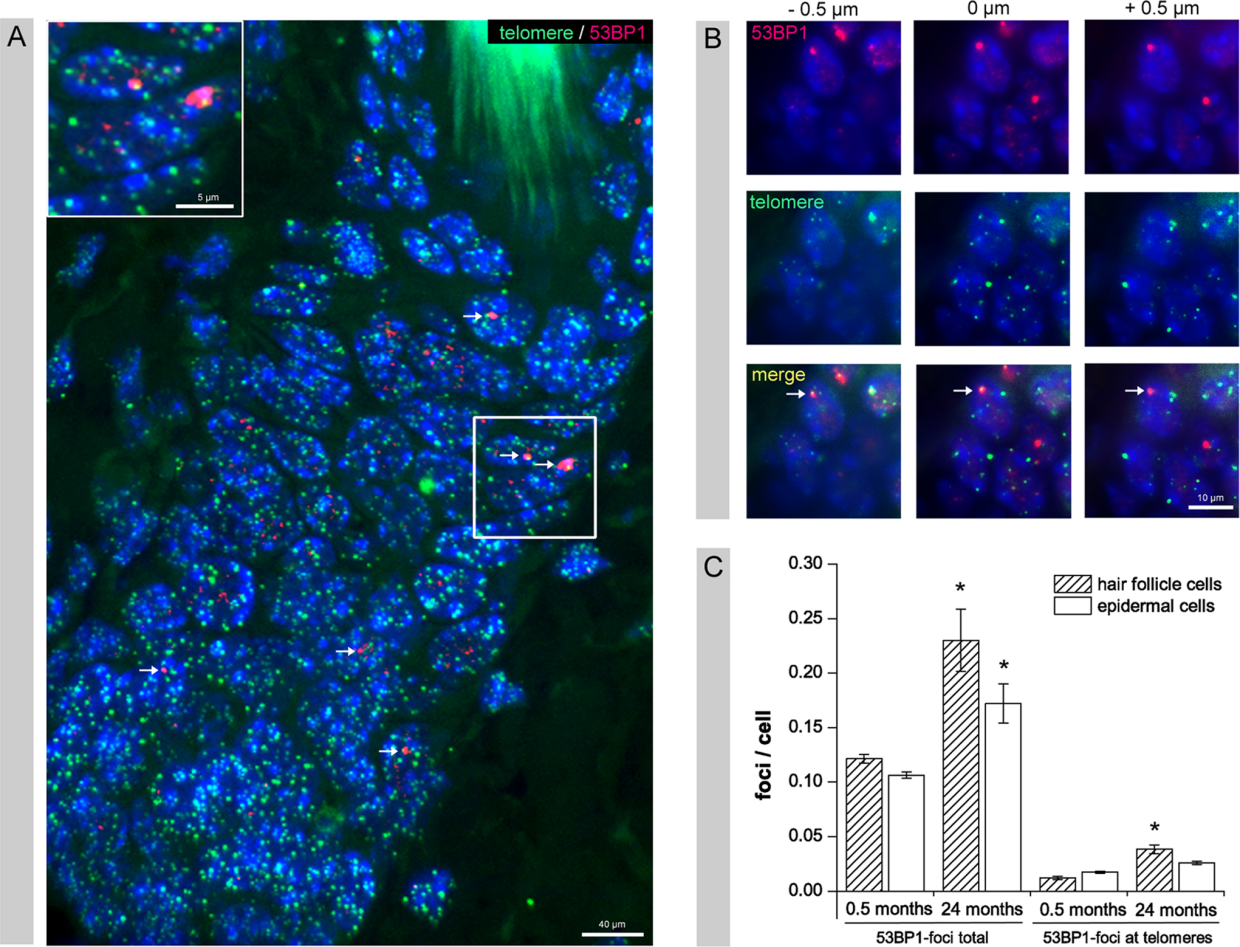
Colocalization of 53BP1-foci with telomeric DNA. (A) IFM micrograph of FISH combined with immunofluorescence to visualize telomeric DNA and 53BP1-foci in skin sections of 24-month-old mice. High numbers of telomeric spots of similar size and intensity (green), but only single, larger 53BP1-foci (red, arrows) were randomly distributed throughout the nuclei. (B) Serial optical section show that only a minority of age-related 53BP1-foci overlaps with telomeric DNA (arrow). (C) Quantitative analysis of the colocalization of 53BP1-foci and telomeric spots, compared to the total number of spontaneous 53BP1-foci, in hair follicle cells and epidermal cells of 3- and 24-month-old mice. For every data point, at least 50 foci were analyzed for colocalization in tissue sections obtained from three different mice, and data are presented as means ±SE. * significant difference to 3-month-old mice.

To determine if telomere shortening is the main source of age-associated DNA damage accumulation, we combined FISH with immunofluorescence to simultaneously visualize telomeric DNA and 53BP1-foci. The fluorescently labeled PNA probe specifically stained telomeric DNA repeat sequences resulting in high numbers of fluorescent spots of similar size and intensity randomly distributed throughout the interphase nuclei (Fig. 2A). Analyzing serial optical sections only a minority of age-related 53BP1-foci showed some overlap with telomeric DNA (Fig. 2B). To quantify this association, we determined the percentage of colocalization between 53BP1-foci and telomeric spots in hair follicle and epidermal cells of 3- and 24-month-old mice (Fig. 2C). We observed an age-related increase of colocalization between 53BP1-foci and telomeric DNA in cells of the hair follicle and to a lesser extent in epidermal cells. In young hair follicle cells only 0.01±0.002 53BP1-foci per cell colocalize with telomeric DNA, but this number increased to 0.04±0.004 foci per hair follicle cell in aged animals (Fig. 2C). However, more than 80% of the 53BP1-foci (0.23±0.005 53BP1-foci in total per hair follicle cell in 24-month-old mice) did not colocalize with telomeric DNA, suggesting that telomere shortening in murine hair follicle cells during physiological aging is not the main cause of DNA damage accumulation.

### Age-related Decrease of DNA Repair Capacity

**Figure 3:**
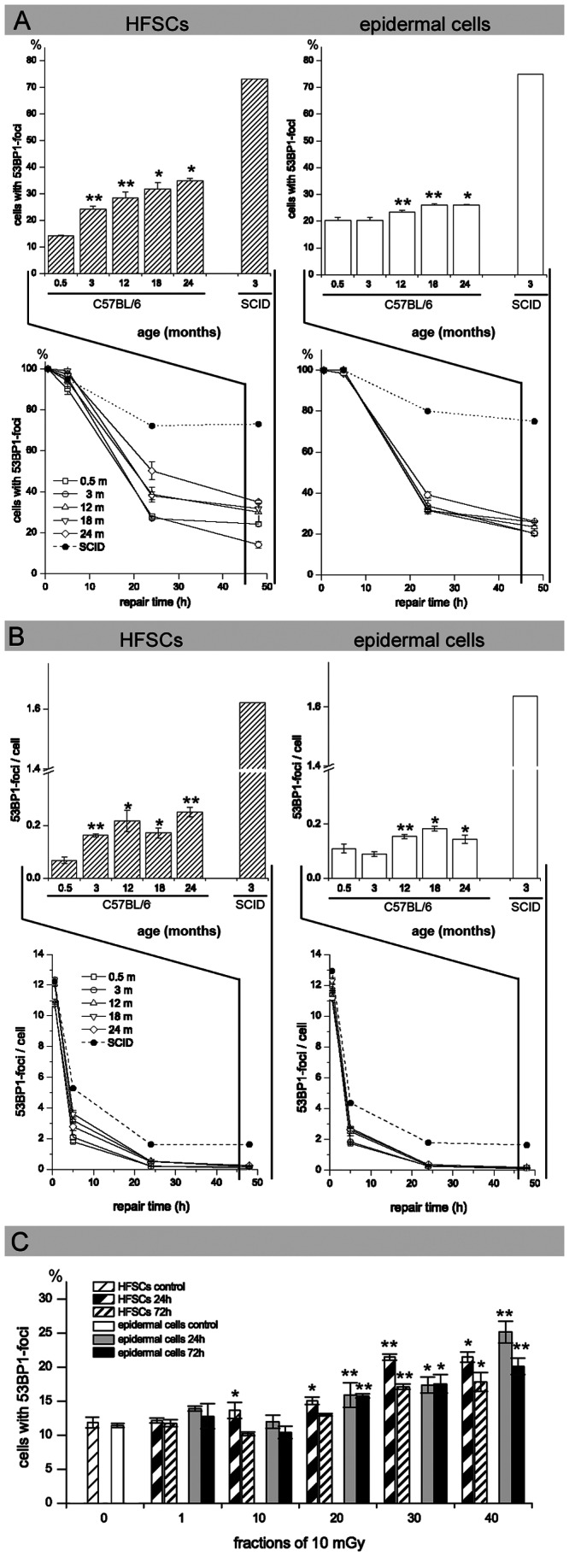
DNA damage response after exposure to ionizing radiation. (A, B) Age-related decrease in DNA repair capacity: DSB repair capacity of HFSCs and epidermal cells was analyzed in skin sections of whole-body irradiated mice by counting cells with 53BP1-foci at defined time-points after 2 Gy irradiation (A). Additionally, the number of 53BP1-foci per cell was counted at defined time-points after 2 Gy irradiation. To analyze the repair capacity independent of the endogenous effect, controls were subtracted from the quantified 53BP1-foci (B). The number of 53BP1-foci is depicted separately for repair-proficient C57BL/6 mice compared to repair-deficient SCID mice. At late repair-times (compare inset for 48 h post-irradiation), HFSCs reveal age-related differences in the number of cells with 53BP1-foci. Data are presented as means from three different experiments ±SE. * significant difference to 0.5-month-old mice; ** significant difference to the younger age-group. (C) DNA damage accumulation during fractionated low-dose radiation: To evaluate the impact of fractionated low-dose radiation (10×, 20×, 30×, and 40× 10 mGy) on cumulative DNA damage, HFSCs and epidermal cells with residual 53BP1-foci were quantified at 24 h and 72 h after the last exposure. Persisting 53BP1-foci levels increase with cumulative radiation doses. Data are presented as means from three different experiments ±SE. * significant difference to unirradiated mice; ** significant difference to the lower dose-group.

We measured the ability of HFSCs and epidermal cells to respond to ionizing radiation within their physiological environment. The DSB repair capacity of HFSCs and epidermal cells was analyzed in skin sections of whole-body irradiated mice by counting cells with 53BP1-foci (A), as well as the total number of 53BP1-foci per cell (B), at defined time points after 2 Gy radiation. In Figure 3 the percentage of cells containing 53BP1-foci (A) and the 53BP1-foci per cell (B) is shown separately for repair-proficient 0.5-, 3-, 12-, 18-, and 24-month-old C57BL/6 mice compared to repair-deficient 3-month-old SCID mice. At 0.5 h after radiation exposure, 100% of the HFSCs and epidermal cells had 53BP1-foci independent of age or repair-proficiency. In SCID mice, the percentage of cells with 53BP1-foci persists at high levels in both HFSCs and epidermal cells (70–80%), reflecting their impaired DSB repair capacity. For repair-proficient C57BL/6 mice the percentage of cells containing 53BP1-foci decreases within 48 h post-irradiation, but at these late repair-times HFSCs reveal age-related differences. Only 14.2±1.6% of 0.5-month-old HFSCs but 35.0±0.85% of 24-month-old HFSCs still had 53BP1-foci at 48 h post-irradiation (Fig. 3A). To reinforce the decline of the repair efficiency with age, we also consider the 53BP1-foci per cell after 2 Gy exposure independent of the endogenous effect (Fig. 3B). Thereby, the number of 53BP1-foci 48 h after radiation increased from 0.07±0.012 foci per cell in 0.5-month-old HFSCs to 0.25±0.018 foci per cell in 24-month-old HFSCs, probably reflecting residual radiation-induced DSBs (Fig. 3B, upper panel). In epidermal cells only minor age-related differences in DSB repair capacity were seen (Fig. 3A; Fig. 3B).This suggests that young HFSCs repair DSBs efficiently and that HFSCs have an age-related reduction in their DSB repair capacity.

### DNA Damage Accumulation during Low-dose Radiation

We aimed to characterize how epidermal stem cells respond to repetitive genotoxic insults induced by low doses of ionizing radiation. To evaluate the impact of fractionated low-dose radiation on cumulative DNA damage, we quantified residual 53BP1-foci at 24 h and 72 h after the last exposure. Fractionated radiation with 10×, 20×, 30× and 40× 10 mGy led to a gradual increase in 53BP1-foci levels in both HFSCs and epidermal cells (Fig. 3C). We observed slightly higher values at 24 h compared to 72 h, probably reflecting continuing repair processes. The fact that not only undifferentiated long-living HFSCs, but also differentiated short-living epidermal cells exhibit increased foci levels suggests that radiation-induced damage in epidermal stem cells may be passed on to downstream differentiated cell lineages. Our data suggest that persisting 53BP1-foci levels increase with cumulative radiation doses, thus mimicking the process of DNA damage accumulation during physiological aging.

### Characterization of Persistent DNA Lesions by TEM

**Figure 4:**
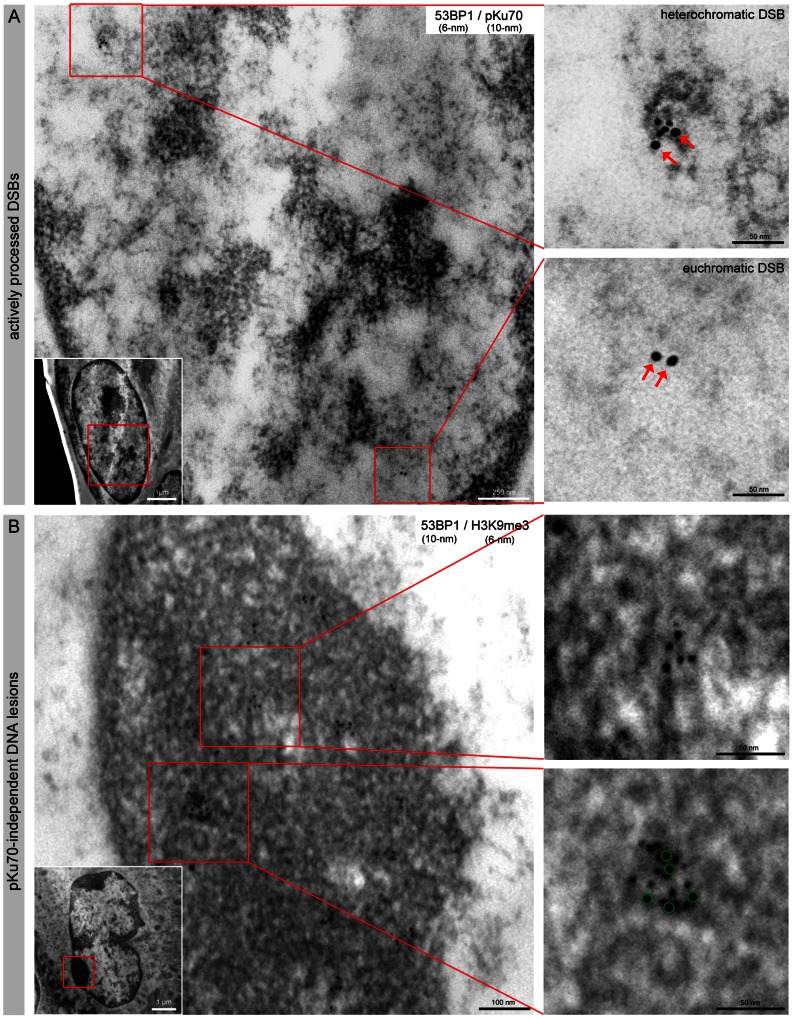
Characterization of radiation-induced DNA lesions by TEM. (A) TEM micrographs of double-labeling of 53BP1 (6-nm) and pKu70 (10-nm) in HFSCs analyzed 0.5 h after 2 Gy irradiation. pKu70 consistently colocalized with 53BP1 in heterochromatic regions, but only pKu70 clusters (without 53BP1) were detected in euchromatic regions. (B) TEM micrographs of double-labeling of 53BP1 (10-nm) and H3K9me3 (6-nm) in HFSCs analyzed 72 h after protracted low-dose radiation (40× 10 mGy). Persistent 53BP1 clusters (green circles at higher magnification) colocalized with the heterochromatin marker H3K9me3 and were localized predominantly in tightly packed heterochromatin (dark grey regions in TEM; compare inset with the overview of the whole nucleus).

By colabeling 53BP1 and pKu70 with gold-beads of different size (6-nm and 10-nm), we characterized the radiation-induced 53BP1-foci in the context of the local chromatin conformation in HFSCs. A striking morphological feature of HFSCs is their irregularly shaped nuclei, compared to epidermal cells with oval shaped nuclei. This feature has previously been described in the bulge cells of murine and human hair follicles (Fig. S2) [25]. At early times after radiation exposure, we observed a constant colocalization of 53BP1 and pKu70 in the periphery of heterochromatic regions (characterized by more relaxed chromatin structure, visualized in TEM as light grey regions), but only isolated pKu70 clusters without any 53BP1 binding in euchromatic regions (Fig. 4A). Similar results were observed by colabeling pKu70 and γH2AX (data not shown). These observations confirm that repair factors such as 53BP1 and γH2AX form foci exclusively at heterochromatic DSBs and support the idea that these components may promote the localized chromatin decondensation that is required for repair in more complex heterochromatin. However, pKu70 is an essential alignment factor and must be present at every break and thus appears to be a reliable marker for actively processed DSBs in euchromatic and heterochromatic domains. In skin samples exposed to protracted low-dose radiation (40× 10 mGy), most of the 53BP1 clusters appear as larger conglomerations of gold-beads that do not colocalize with pKu70 or DNA-PKcs. These 53BP1 clusters consistently colocalize with the heterochromatin marker H3K9me3 and localize predominantly in tightly packed heterochromatin (dark grey regions in TEM; compare inset with the overview of the whole nucleus) (Fig. 4B).

**Figure 5:**
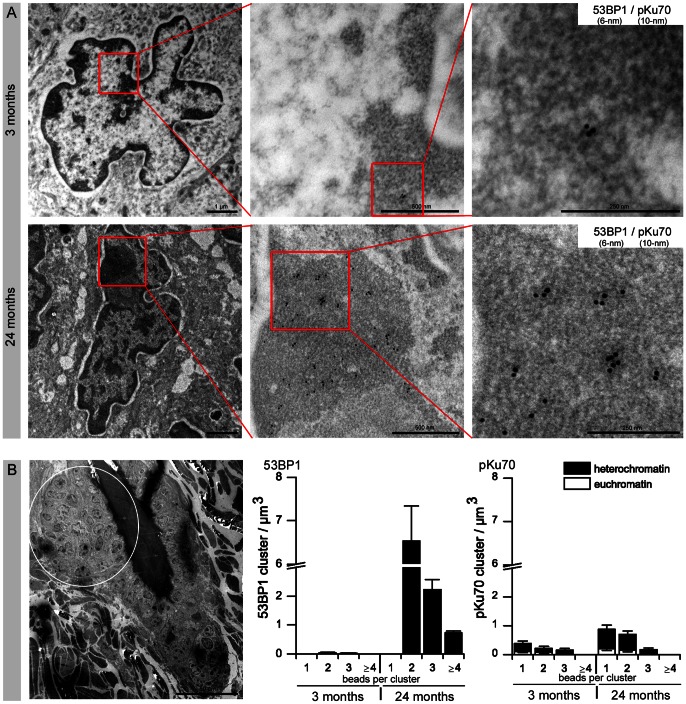
Characterization of age-related DNA lesions by TEM. (A) TEM micrographs of double-labeling of 53BP1 (6-nm) and pKu70 (10-nm) in HFSCs of 3- and 24-month-old mice. In skin samples of aged mice, but not young mice, multiple 53BP1 clusters of 2–6 gold-beads are scattered throughout the highly condensed heterochromatin of HFSCs. These persistent 53BP1 clusters did not colocalize with pKu70. (B) The area highlighted by a circle in the TEM micrograph indicates the region studied. Quantitative analysis of the 53BP1 and pKu70 cluster distribution in eu- and heterochromatin of HFSCs in skin sections of 3- and 24-month-old animals. Data are presented as means from three different experiments ±SE. * significant difference to 3-month-old mice.

We performed analogous TEM analysis to characterize the age-related 53BP1-foci. In skin samples of 24-month-old mice, but not 3-month-old mice, we observed multiple 53BP1 clusters of 2–6 gold-beads scattered throughout the highly condensed heterochromatin of the HFSCs, and these 53BP1 clusters did not colocalize with pKu70 or DNA-PKcs (Fig. 5A). In the TEM image of Fig. 5B the area highlighted by a circle indicates the region studied. Quantitative analysis of the 53BP1 cluster distribution in skin tissue of 3- and 24-month-old animals showed increased levels of 53BP1 clusters exclusively in the compact heterochromatin of aged HFSCs (Fig. 5B). Significantly, low levels of pKu70 clusters were detected in euchromatic and heterochromatic compartments of HFSCs in both 3- and 24-month-old animals, suggesting that ROS-induced DSBs can arise in both forms of chromatin. However, aged animals had higher pKu70 levels in their HFSCs, which may reflect an age-related impairment of their DSB repair capacity. The TEM characterization of the focal accumulation of 53BP1 in HFSCs showed single large 53BP1 clusters in irradiated skin, but multiple small 53BP1 clusters in aged skin. These two kinds of 53BP1 clusters colocalized with the heterochromatin marker H3K9me3, but not pKu70 or pDNA-PKcs, and were both found primarily in highly compacted heterochromatin.

### Resistance of HFSCs to DNA Damage-induced Cell Death

**Figure 6:**
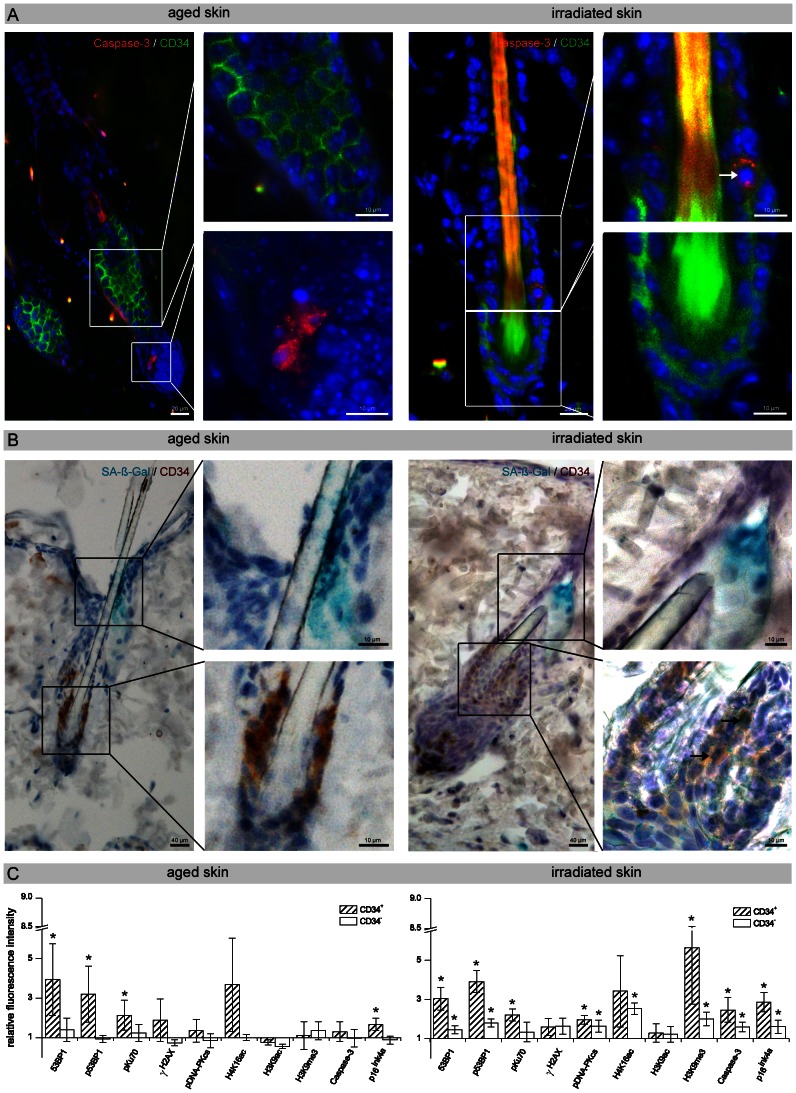
Biological outcome of HFSCs in aged and irradiated skin. (A) IFM micrographs of Caspase-3 staining of skin sections derived from aged (24-months-old) and low-dose irradiated animals (40× 10 mGy). Aged skin revealed slightly increased levels of apoptotic cells (red), but not in the CD34**^+^** HFSCs (green) of the bulge region. After protracted low-dose radiation, slightly higher levels of apoptotic cells were observed, and even some CD34**^+^** HFSCs undergo apoptosis (arrow in the enlarged image). (B) Micrographs of the histochemical detection of SA-β-gal activity in skin sections derived from aged (24-months-old) and low-dose irradiated animals (40× 10 mGy). SA-β-gal staining of aged skin revealed an increase of the blue-dyed precipitate in the upper isthmus and infundibulum of the hair follicle, but not in CD34**^+^** HFSCs of the bulge region (brown). In irradiated skin, some CD34**^+^** HFSCs stained positive for SA-β-gal (two arrows in the enlarged lower image). Sporadic staining of the lumen of sebaceous ducts was also seen (enlarged upper image). (C) Analysis of the DNA damage response by flow-assisted cytometry. Relative fluorescence intensity of repair proteins, histone modifications, apoptosis and senescence markers in CD34**^+^** and CD34**^–^** cells derived from aged (24-month-old) and irradiated (40× 10 mGy; 72 h) skin normalized to unirradiated young skin (3-month-old). Data are presented as means from three different experiments ±SE. * significant difference compared with unirradiated young skin.

To define the functional consequences of DNA damage accumulation, we assessed the induction of apoptosis by immunostaining for the cleaved form of Caspase-3. In the absence of radiation-induced genotoxic stress, both young and old skin exhibited comparably low levels of apoptosis (epidermal cells: 0.46±0.16%; HFSCs: 0.61±0.12% Caspase-3 positive cells) (Fig. S3A; Fig. S3C). Aged skin displayed slightly increased levels of apoptotic cells in the basal epidermal layers, but not in the CD34^+^ epidermal stem cells (epidermal cells: 0.68±0.10%; HFSCs: 0.65±0.18% Caspase-3 positive cells), suggesting that aged HFSCs are rather resistant to DNA damage-induced apoptosis (Fig. 6A, left panel). After protracted low-dose radiation, however, we observed slightly higher levels of apoptotic epidermal cells and even apoptotic CD34^+^ HFSCs in response to radiation-induced DNA damage (Fig. 6A, right panel). The apoptotic index increased to 1.03±0.15% positive epidermal cells and even to 2.41±0.23% positive HFSCs. It is possible that aged HFSCs do not undergo apoptosis, but instead undergo cellular senescence to preserve the architecture of the epidermis. To determine the level of senescence during physiological aging, young and old skin was examined for the expression of senescence-associated β-galactosidase (SA-β-gal) activity. Young skin exhibited very low levels of senescent cells (epidermal cells: 1.88±0.46%; HFSCs: 1.40±0.49% SA-ß-gal positive cells) (Fig. S3B, S3C) and old tissues showed a slightly higher fraction of SA-β-gal positive HFSCs (3.45±0.51%) (Fig. 6B, left panel; Fig. S3C). After low-dose radiation, differentiated epidermal cells of the epidermis and even HFSCs showed increased SA-β-gal and p16^ink4a^ activity (epidermal cells: 11.42±2.15%; HFSCs: 17.62±3.75% SA-ß-gal positive cells) (Fig. 6B, right panel; Fig. S3C; Fig. S3D). Hence, our results suggest that HFSCs do not undergo apoptosis or cellular senescence during the physiological aging process, but after protracted low-dose radiation the number of apoptotic and senescent HFSCs increases considerably.

### Analysis of the DNA Damage Response by Flow-assisted Cytometry

We used phospho-specific antibodies that detect activated repair proteins in combination with euchromatic and heterochromatic histone modifications to characterize the mechanisms of DDR in aged and irradiated HFSCs by flow-assisted cytometry. This multiparametric analysis was related to the induction of apoptosis or senescence (Fig. 6C). Single-cell suspensions were isolated from skin tissues of 3- and 24-month-old animals and from low-dose irradiated and unirradiated mice. In CD34**^+^** (tissue-specific stem cells) and CD34**^−^** cell populations (differentiated cells) isolated from aged and irradiated epidermis, the expression of multiple parameters were simultaneously measured as the relative integrated fluorescence intensity compared to unirradiated skin of 3-month-old animals. In CD34**^+^** HFSCs isolated from aged skin, the repair proteins 53BP1, p53BP1, and pKu70 were significantly upregulated, likely signaling the presence of progressive oxidative DNA damage. Aged HFSCs were also characterized by slightly increased H4K16ac levels, a characteristic epigenetic alteration associated with aging. This increased DDR was not associated with elevated Caspase-3 levels indicating that aged HFSCs do not undergo DNA damage-induced apoptosis; but slightly increased p16**^ink4a^** levels suggest the existence of senescent CD34^+^ HFSCs. However, in more differentiated CD34**^−^** cell populations no significant differences between young and old epidermis was observed regarding the expression of repair factors, histone modifications, or DNA damage-induced cell death (Fig. 6C, left panel). CD34**^+^** HFSCs obtained from skin tissue exposed to protracted low-dose radiation had significantly increased expression levels of 53BP1, p53BP1, pKu70, and pDNA-PKcs compared to unirradiated tissue, probably reflecting constitutive DDR triggered by repetitive genotoxic insults (Fig. 6C, right panel). These irradiated HFSCs were characterized by increased H4K16ac levels, probably reflecting the chromatin relaxation upon DNA damage, and by significantly increased H3K9me3 levels. Methylation of H3K9 provides a surface for association of the chromodomain of heterochromatin protein 1 (HP1) to promote binding to heterochromatin and is, therefore, generally associated with transcriptional repression. In physiologically aged HFSCs, we observed slightly increased p16^ink4a^ levels, reflecting that more HFSCs have entered senescence in old than in young animals. Low-dose irradiated HFSCs had a significant increase in Caspase-3 and p16^ink4a^ levels, suggesting that repetitive genotoxic stress increases both DNA damage-induced apoptosis and cellular senescence (Fig. 6C, right panel).

## Discussion

The DNA damage theory of aging postulates that the main cause of the functional decline associated with aging is the accumulation of DNA damage, ensuing cellular alterations and disruption of tissue homeostasis [1]. Using an in-vivo model with aged mice to analyze epidermal stem cells within their physiological niche, we observed an increasing amount of 53BP1-foci in HFSCs during the natural aging process that was not related to telomere shortening or dysfunction. A similar increase in 53BP1-foci was observed during repeated low-dose radiation suggesting a substantial accumulation of DSBs. TEM visualization showed multiple small 53BP1 clusters in aged HFSCs, but single large 53BP1 clusters in irradiated HFSCs, and these clusters were found exclusively in highly compacted heterochromatin and were associated with heterochromatic histone modifications. These persisting clusters did not colocalize with pKu70. Referring to the central role as a core component of the NHEJ, we suggest that these lesions are not actively processed DSBs, but may reflect permanent chromatin rearrangements caused by the repair or misrepair of DSBs. Aged HFSCs characterized by multiple small 53BP1 clusters showed neither apoptosis nor senescence. Irradiated HFSCs, however, were characterized by single, but more complex 53BP1 clusters and signaling pathways for apoptosis or senescence were triggered. This may result in a faster depletion of stem cells and contribute to reduced tissue regeneration and accelerated aging. We hypothesize that the profound structural differences between 53BP1 clusters induced by intrinsic or extrinsic DNA damage may reflect the complexity of the initial genotoxic insult and ultimately lead to the different biological outcomes.

Beyond the function of packaging DNA in the nucleus, chromatin organization can also convey epigenetic information that contributes to the fine-tuning of gene expression. Chromatin structure is subjected to substantial rearrangements during the DDR, and may facilitate repair by promoting DNA accessibility in the vicinity of damage. After the repair of DNA lesions, the chromatin structure must be faithfully restored to preserve the epigenetic information. Depending on the level of compaction or state of the chromatin, euchromatic and heterochromatic DNA are characterized by specific patterns of histone modifications that regulate chromatin accessibility and distinct transcriptional functions. The re-establishment of the epigenetic status in a damaged chromatin region may depend on the intensity of damage, and may be particularly critical in specialized chromatin regions such as heterochromatin domains.

Ionizing radiation can damage DNA through direct action, producing single- and double-strand breaks in the DNA double helix, as well as indirect effects by generating ROS in the cells. In previous TEM studies we established the immunogold-labeling technique specifically for the phosphorylated form of Ku70 (pKu70, phosphorylated at serine 6), which directly binds to broken DNA ends [15], [16]. Significantly, most of the pKu70 clusters consisted of two beads, in which the observed distance of about 15 nm between the two pKu70 beads conforms to the known three-dimensional structure of the Ku70-Ku80 complex [5], [26]. Moreover, analyzing the repair kinetics of radiation-induced DSBs after single-dose exposure to 6 Gy, we observed pKu70 clusters consisting of up to 10 or 12 beads in heterochromatic domains at late repair times, which may reflect multiple breaks in close proximity caused by the direct or indirect effect of ionizing radiation in highly compacted DNA [16]. In experiments with densely ionizing charged particles, we observed complex DNA lesions with hundreds of pKu70 and 53BP1 beads along the initial linear particle trajectories representing the primary sites of damage induction. These findings show that ionizing radiation can lead to considerably more complex DNA damage that is more difficult to repair than the simple and diffuse damage caused by oxidative stress. In these previous TEM analyses, we observed an increase in huge 53BP1 clusters (up to 30–60 gold beads) in heterochromatic domains at 48 h and 72 h after radiation, occasionally colocalizing with γH2AX. However, these huge 53BP1 clusters did not colocalize with pKu70 or DNA-PKcs, indicating that these lesions were not actively processed by NHEJ [15]. These 53BP1 clusters were detectable at nearly constant levels even 1 week after radiation suggesting that these lesions may represent permanent chromatin rearrangements due to repair or misrepair of radiation-induced DSBs [15]. Translating these results to our current study, we assume that the extent of the initial damage in the local hit region of DNA likely influences the degree of chromatin alteration. We speculate that multiple damaged DNA sites induced by ionizing radiation cause severe disruptions of the chromatin structure, and may have profound effects on the biological outcome of the affected cells.

Our results are in accordance with the model proposed by P. Jeggo and coworkers that ATM (ataxia telangiectasia mutated) signaling plays a major role in modifying chromatin structure in the vicinity of DSBs. ATM's role in the repair of heterochromatic DSBs involves the direct phosphorylation of KAP-1, a key heterochromatin formation factor [27], [28], [29], [30]. Mediator proteins such as 53BP1, which are also essential for the repair of heterochromatic DSBs, promote the retention of activated ATM at DSBs, concentrating the phosphorylation of KAP-1 at heterochromatic DSBs, and thereby modulating chromatin structures surrounding the break site [30].

During physiological aging, DNA is continuously exposed to intracellular oxidants, particularly the by-products of metabolic activity, that lead to progressive oxidative DNA damage. It is estimated that these oxidants generate approximately 5000 single-strand breaks (SSBs) in the average cell in the human body every day [31]. About 99% of SSBs are repaired by essentially error-free mechanisms, but the remainders are converted to DSBs, predominantly during DNA replication [18]. The NHEJ pathway is error-prone, often resulting in deletion of a few base pairs. This leads to an accumulation of permanent DNA damage that is considered to be the primary cause of cellular aging and senescence. However, our findings suggest that age-associated DNA damage leads to smaller structural defects in heterochromatic domains, as visualized by TEM, and thus may have less profound effects on the biological outcome.

Incomplete or incorrect restoration of the original, undamaged chromatin state may affect epigenetic regulatory processes involved in gene expression control. Considering the critical importance of chromatin organization for correct gene expression, proper restoration of epigenetic patterns following DNA damage is likely crucial to avoid disturbances in transcriptional programs. Perturbations in epigenetic regulatory functions affect the vast majority of nuclear processes, including gene transcription and silencing, DNA replication and repair, cell cycle progression, telomere structure and function, and are known to be associated with physiological aging [32], [33].

Collectively, our results suggest that progressive DNA damage induced by endogenous or exogenous insults results in different degrees of pKu70-independent chromatin alterations but not in the accumulation of actively processed DSBs. These findings support the hypothesis that aging might be largely the remit of structural changes to chromatin potentially leading to epigenetically induced transcriptional deregulation.

## Supporting Information

Figure S1 **Age-related DNA damages in HFSCs.** IFM micrographs of radiation-induced 53BP1-foci (red) in Dapi stained nuclei (blue) of CD34-positive HFSCs (green) of 0.5- and 24-month-old mice.
(TIF)

Figure S2 **Characterization of HFSCs by TEM.** TEM micrographs of CD34 immunogold-labeled skin sections. HFSCs characterized by their irregularly shaped nuclei, are located in the bulge region of the hair follicle (left and middle micrographs) (upper panel). These HFSCs can also be identified by CD34 gold-beads (red circles) exculsively found at the cytoplasmic membrane of the HFSC (right micrograph). In contrast, epidermal cells with normal oval shaped nuclei are located in the epidermal layers. These epidermal cells show no CD34 gold-beads at the cytoplasmic membrane (lower panel).
(TIF)

Figure S3 **Biological outcome.** (A) IFM micrograph of Caspase-3/CD34 double-stained skin sections derived from young (2-month-old) mice. Young skin revealed apoptotic (red) cells neither in the bulge region (CD34^+^ HFSCs (green)) nor in the epidermal layers. (B) Micrograph of the histochemical detection of SA-β-gal activity in skin sections derived from young (2-month-old) animals. Young skin showed an age-independent SA-ß-Gal staining in the sebaceous gland but not in CD34**^+^** HFSCs (brown) or in epidermal cells. (C) Quantification of Caspase-3 (left panel) and SA-ß-gal (right panel) positive CD34^+^ HFSCs and epidermal cells of young (2-month-old), aged (24-month-old) and low-dose irradiated mice (40× 10 mGy; 72 h). The apoptotic and senescence index shows a clear increase in both cell types after fractionated low-dose irradiation. Data are presented as means from three different experiments ±SE. * significant difference to 2-month-old mice. (D) Micrographs of the histochemical detection of p16^ink4a^ activity in skin sections derived from young (2-month-old) and low-dose irradiated animals (40× 10 mGy; 72 h). p16^ink4a^ staining of irradiated skin revealed an increase of stained cells (brown) in the epidermal layers and in the hair follicle, compared to the young control.
(TIF)
