# Supplementary figures and images for: Accumulation of DNA Damage-Induced Chromatin Alterations in Tissue-Specific Stem Cells: The Driving Force of Aging?
Source: PLoS One. 2013 May 17;8(5):e63932. doi: 10.1371/journal.pone.0063932 (PMC3656879; doi:10.1371/journal.pone.0063932)

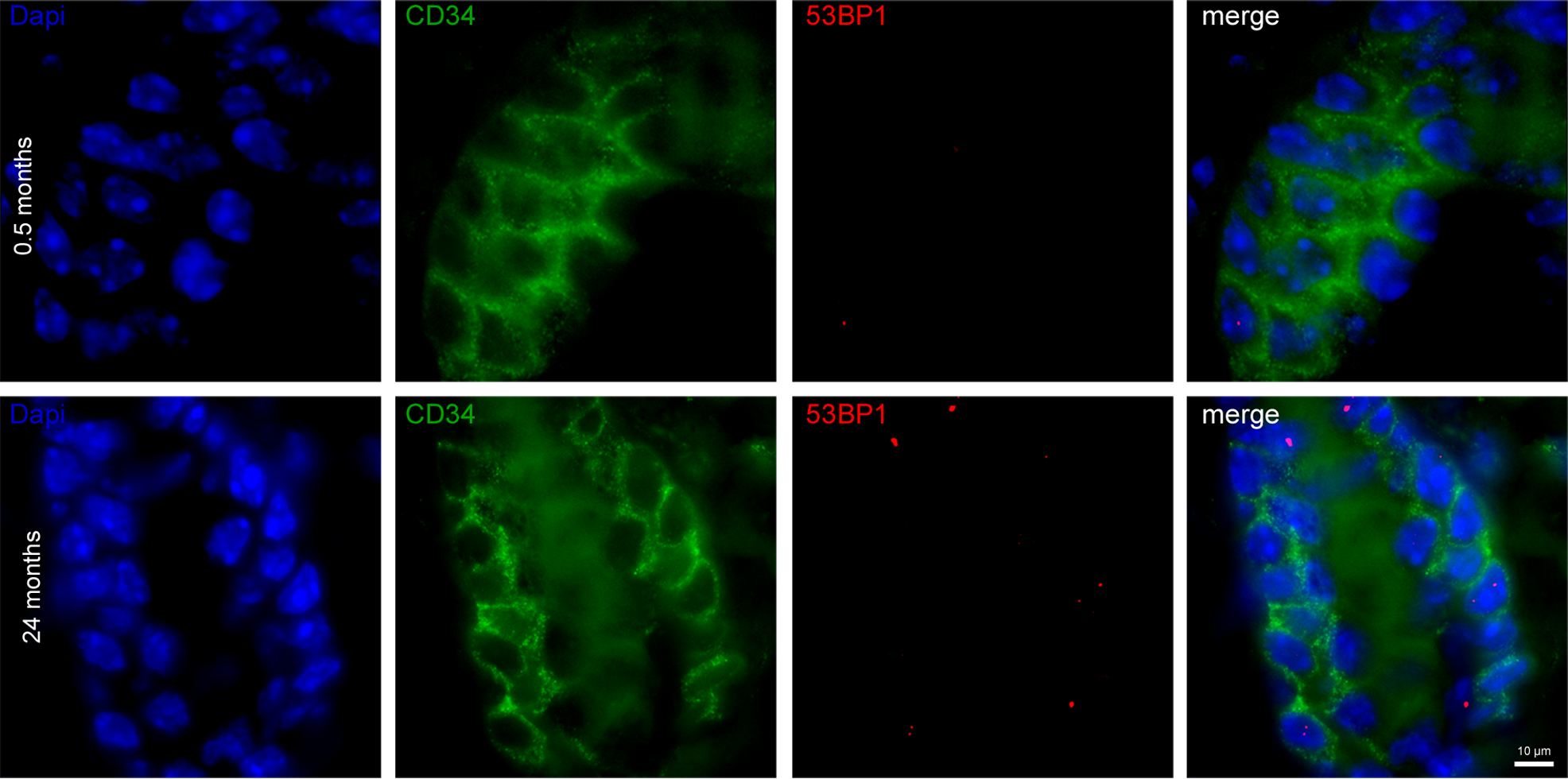

Supplement: Figure S1 — Age-related DNA damages in HFSCs. IFM micrographs of radiation-induced 53BP1-foci (red) in Dapi stained nuclei (blue) of CD34-positive HFSCs (green) of 0.5- and 24-month-old mice. (TIF) [file pone.0063932.s001.tif]

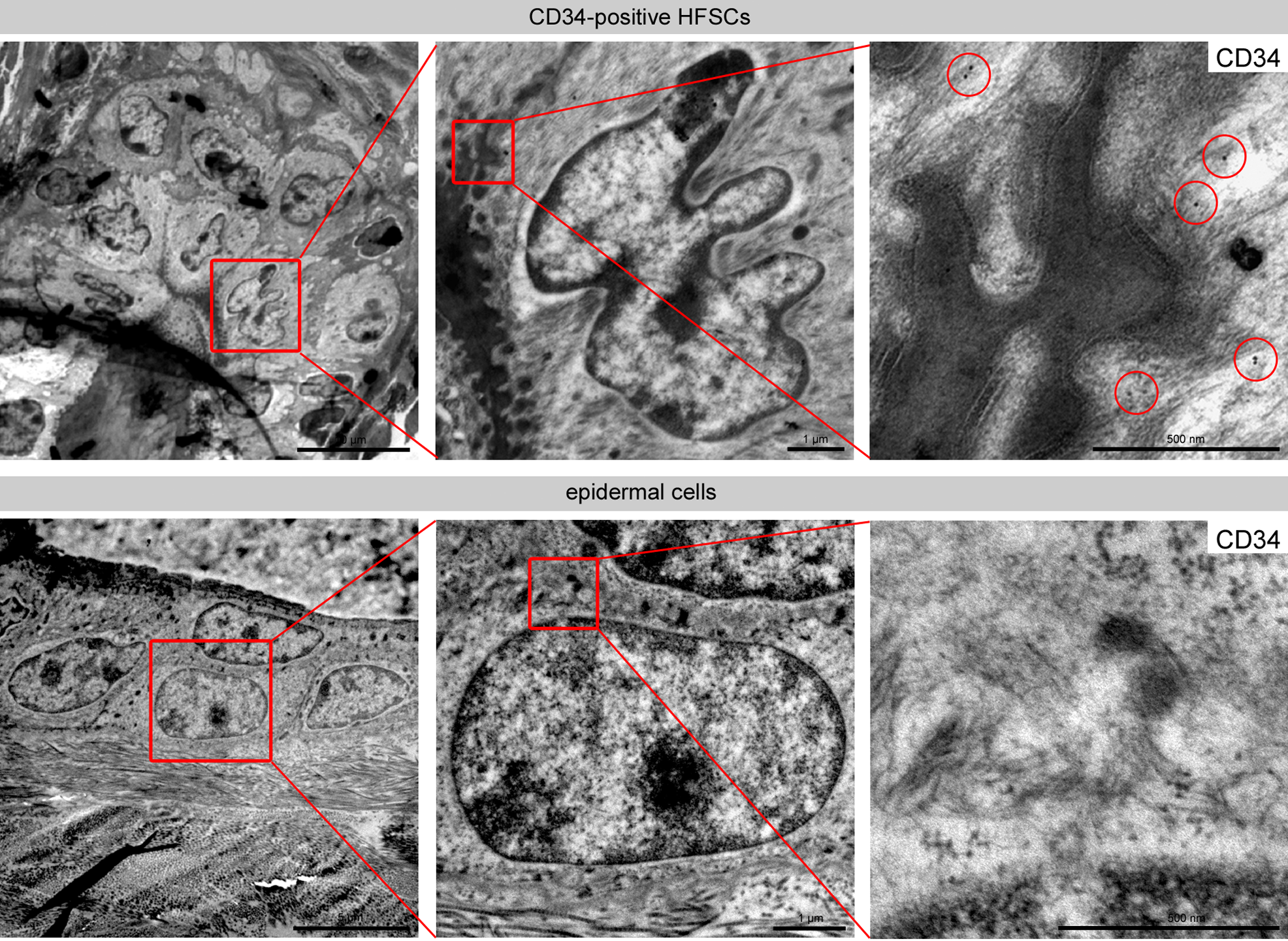

Supplement: Figure S2 — Characterization of HFSCs by TEM. TEM micrographs of CD34 immunogold-labeled skin sections. HFSCs characterized by their irregularly shaped nuclei, are located in the bulge region of the hair follicle (left and middle micrographs) (upper panel). These HFSCs can also be identified by CD34 gold-beads (red circles) exculsively found at the cytoplasmic membrane of the HFSC (right micrograph). In contrast, epidermal cells with normal oval shaped nuclei are located in the epidermal layers. These epidermal cells show no CD34 gold-beads at the cytoplasmic membrane (lower panel). (TIF) [file pone.0063932.s002.tif]

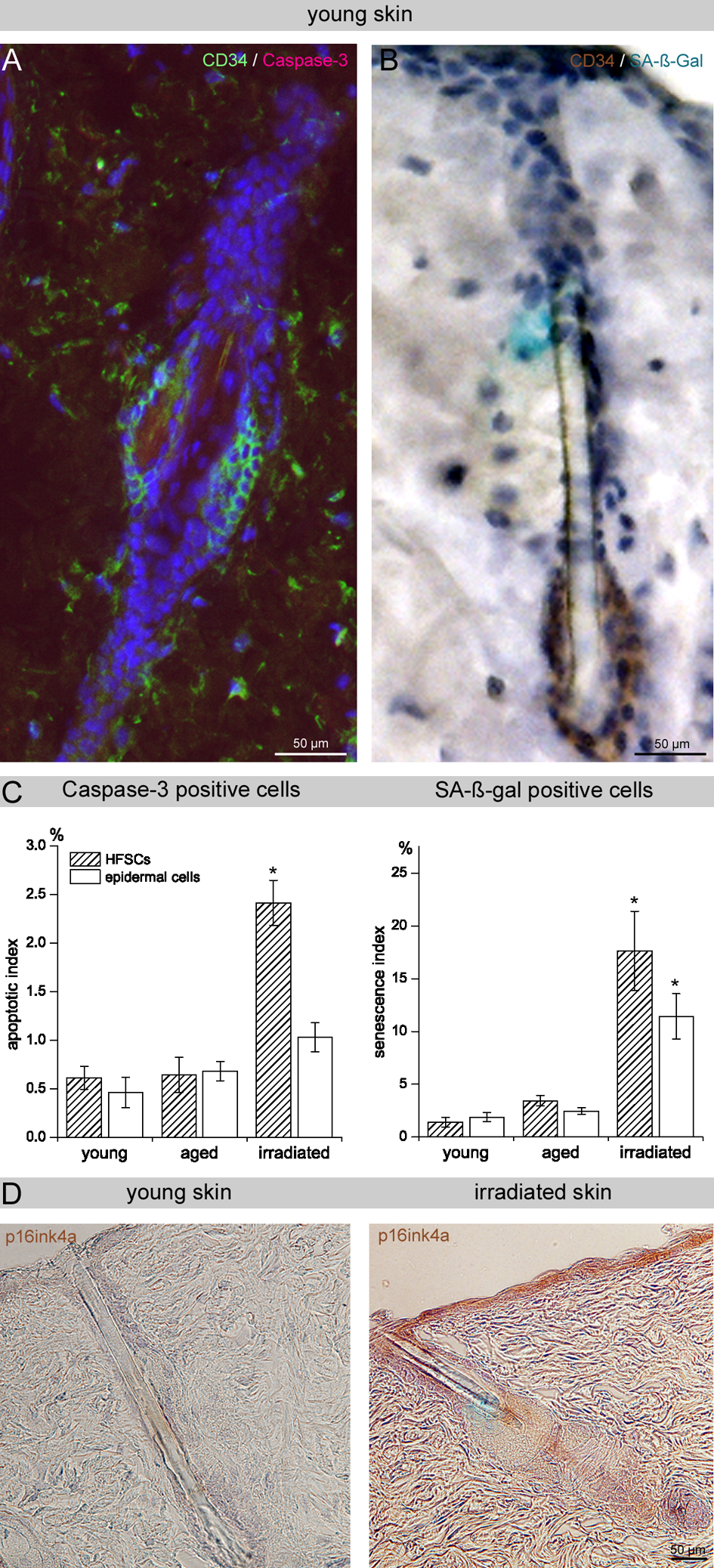

Supplement: Figure S3 — Biological outcome. (A) IFM micrograph of Caspase-3/CD34 double-stained skin sections derived from young (2-month-old) mice. Young skin revealed apoptotic (red) cells neither in the bulge region (CD34+ HFSCs (green)) nor in the epidermal layers. (B) Micrograph of the histochemical detection of SA-β-gal activity in skin sections derived from young (2-month-old) animals. Young skin showed an age-independent SA-ß-Gal staining in the sebaceous gland but not in CD34+ HFSCs (brown) or in epidermal cells. (C) Quantification of Caspase-3 (left panel) and SA-ß-gal (right panel) positive CD34+ HFSCs and epidermal cells of young (2-month-old), aged (24-month-old) and low-dose irradiated mice (40× 10 mGy; 72 h). The apoptotic and senescence index shows a clear increase in both cell types after fractionated low-dose irradiation. Data are presented as means from three different experiments ±SE. * significant difference to 2-month-old mice. (D) Micrographs of the histochemical detection of p16ink4a activity in skin sections derived from young (2-month-old) and low-dose irradiated animals (40× 10 mGy; 72 h). p16ink4a staining of irradiated skin revealed an increase of stained cells (brown) in the epidermal layers and in the hair follicle, compared to the young control. (TIF) [file pone.0063932.s003.tif]
